# Gene expression patterns associated with p53 status in breast cancer

**DOI:** 10.1186/1471-2407-6-276

**Published:** 2006-12-06

**Authors:** Melissa A Troester, Jason I Herschkowitz, Daniel S Oh, Xiaping He, Katherine A Hoadley, Claire S Barbier, Charles M Perou

**Affiliations:** 1 Division of Biostatistics and Epidemiology, School of Public Health and Health Sciences, University of Massachusetts Amherst, Amherst, MA, USA; 2 Lineberger Comprehensive Cancer Center, University of North Carolina at Chapel Hill, Chapel Hill, NC, USA; 3 Curriculum in Genetics and Molecular Biology, University of North Carolina at Chapel Hill, Chapel Hill, NC, USA; 4 Department of Genetics, University of North Carolina at Chapel Hill, Chapel Hill, NC, USA; 5 Department of Pathology and Laboratory Medicine, University of North Carolina at Chapel Hill, Chapel Hill, NC, USA

## Abstract

**Background:**

Breast cancer subtypes identified in genomic studies have different underlying genetic defects. Mutations in the tumor suppressor p53 occur more frequently in estrogen receptor (ER) negative, basal-like and HER2-amplified tumors than in luminal, ER positive tumors. Thus, because p53 mutation status is tightly linked to other characteristics of prognostic importance, it is difficult to identify p53's independent prognostic effects. The relation between p53 status and subtype can be better studied by combining data from primary tumors with data from isogenic cell line pairs (with and without p53 function).

**Methods:**

The p53-dependent gene expression signatures of four cell lines (MCF-7, ZR-75-1, and two immortalized human mammary epithelial cell lines) were identified by comparing p53-RNAi transduced cell lines to their parent cell lines. Cell lines were treated with vehicle only or doxorubicin to identify p53 responses in both non-induced and induced states. The cell line signatures were compared with p53-mutation associated genes in breast tumors.

**Results:**

Each cell line displayed distinct patterns of p53-dependent gene expression, but cell type specific (basal vs. luminal) commonalities were evident. Further, a common gene expression signature associated with p53 loss across all four cell lines was identified. This signature showed overlap with the signature of p53 loss/mutation status in primary breast tumors. Moreover, the common cell-line tumor signature excluded genes that were breast cancer subtype-associated, but not downstream of p53. To validate the biological relevance of the common signature, we demonstrated that this gene set predicted relapse-free, disease-specific, and overall survival in independent test data.

**Conclusion:**

In the presence of breast cancer heterogeneity, experimental and biologically-based methods for assessing gene expression in relation to p53 status provide prognostic and biologically-relevant gene lists. Our biologically-based refinements excluded genes that were associated with subtype but not downstream of p53 signaling, and identified a signature for p53 loss that is shared across breast cancer subtypes.

## Background

The tumor suppressor p53 is mutated in 30% of breast cancers [1], but rates of p53 mutation vary depending upon the subtype of breast cancer. For example, p53 mutations are found more frequently in aggressive estrogen receptor (ER)-negative breast cancers [1], and have been shown to correlate with breast cancer subtype in gene expression studies [2] and in a population-based study [3]. Genetic abnormalities such as amplified HER2/ERBB2 [1] and aneuploidy [4] are also frequently associated with p53 mutation status. These correlations suggest intrinsic heterogeneity of p53 signaling across breast cancer subtypes.

Gene expression studies can help to characterize breast cancer heterogeneity. Previous *in vitro *studies of gene expression have demonstrated that cell line models of luminal breast cancers show a strong stress response following chemotherapeutic treatment, with notable changes in p53-regulated genes such as p21 (Cip1). The same magnitude of p53-regulated responses was not observed for cell line models of basal-like breast cancer [5]. Inherent differences in p53 signaling and function according to cell type of origin could account for the association between rates of p53 mutation and breast cancer subtype. In this study, we engineered isogenic cell line pairs with and without p53 function using RNA interference (RNAi) and examined the stress responses of parent and RNAi-transduced cell lines. Our aim was to assess how variation in cell line backgrounds alters the effects of p53 loss. We also aimed to identify a common response to p53 loss that is shared by most breast cancers. Thus, we compared the lists of p53-responsive genes *in vitro *to gene lists derived from *in vivo *breast tumor data to identify a set of common p53 responsive genes. The biological relevance of this common p53 signature was assessed by using this gene list to predict outcomes on independent test data sets of breast cancer patients.

## Methods

### Cells and culture conditions

Two hTERT immortalized Human Mammary Epithelial (HME) cell lines (ME16C and HME-CC) and two established breast cancer cell lines (MCF-7 and ZR-75-1) were cultured as described previously [5]. A mitochondrial dye conversion (MTT) assay was used to measure cell line responses to 36 h of treatment with 0 – 10 μM doxorubicin hydrochloride (DOX) [5].

Short hairpin RNAs (RNAi) against p53 were constructed using a 19-mer sequence (GACTCCAGTGGTAATCTAC) described previously [6], but using the pSUPER.RETRO.puro vector with stuffer (Oligo Engine, Seattle, WA). A version of this vector containing two mismatches within the 19-mer sequence (GACTCCGGTTGTAATCTAC) was also prepared as a mismatch control. HEK-293T cells were transfected with 10 micrograms each of pSUPER.RETRO.puro vector, pVpack-GP (Stratagene), and pVpack-Ampho (Stratagene) using Lipofectamine Reagent and PLUS Reagent (Invitrogen). Supernatants containing replication-incompetent retrovirus were collected 48 hours after transfection and applied to all four cell lines. Stable populations of cell lines expressing p53-RNAi or mismatch-RNAi were selected for two weeks in 1 μg per mL puromycin.

### Western blots

Cells were treated for 24 h with 1 μM DOX, and cell free extracts, protein quantitation, and denaturation were as described previously [5]. Forty μg of protein were electrophoresed on a 4–20% Tris-HCl Criterion precast gel (BioRad) and transferred to a Hybond-P membrane (Amersham Biosciences) by electroblotting. The blots were probed with antibodies against p53 (Santa Cruz; D01) and β-actin (Abcam, AC-15) and then with anti-mouse IgG horseradish peroxidase linked whole antibody from sheep (Amersham). Enhanced chemiluminescence was used for detection (SuperSignal West Pico Chemiluminescent Substrate, Pierce).

### Microarray experiments

Cell lines were grown, treated for 12, 24, or 36 h with DOX at the IC50 concentration, and harvested using a previously described protocol [5]. Feeding control (sham) and reference mRNA samples were prepared as described previously [5]. Cy3- and Cy5-labeled cDNAs were synthesized from control or treated cell line mRNA, respectively, according to a direct labeling protocol (Agilent Technologies), and were hybridized to Human 1A oligonucleotide arrays (Agilent Technologies). All microarray raw data tables have been deposited in the Gene Expression Omnibus under the accession number of GSE3178 (submitter C. Perou).

### Identification of p53-dependent DOX-response signature from microarray data

For all comparisons, *in vitro *and *in vivo *as described below, genes that were significantly different in expression were identified using a 2-class, unpaired Significance Analysis of Microarrays (SAM) [7]; for the SAM analysis, the data were first filtered to exclude genes that did not have mean signal intensity greater than twice the median background value for both the red and green channel in at least 70% of the experiments. The SAM delta values were adjusted to obtain the largest gene list that gave a false discovery rate of less than 5%. Using the SAM-derived gene lists, average linkage hierarchical cluster analysis was conducted using Pearson correlation in the Cluster program and the data were visualized in Treeview [8,9]. EASE, the Expression Analysis Systematic Explorer was used to identify enriched biological themes in gene lists [10].

Each cell line was examined for p53 response in both untreated and DOX-treated states. To identify the gene expression effects of p53 loss in DOX-treated cells (p53-induced state), parent cell lines treated with DOX (n = 3 for each cell line) were compared to RNAi-transductants treated with DOX (n = 3 for each cell line). To identify the gene expression effects of p53 loss in the absence of DOX treatment, sham-treated parent cell lines (wildtype p53) were compared to sham-treated RNAi-transductants (n = 3 for both treatment groups in each cell line). However, to derive a list of genes that were differentially expressed in both *in vitro *and the *in vivo *data sets, the common p53 response across all four cell lines was the most relevant. Thus, we also performed an analysis comparing all RNAi-transduced cell line experiments (n = 24) to all parent cell line experiments (n = 24). The resulting list represented the common response to p53 loss across cell lines.

To identify the gene expression signature associated with p53 *in vivo*, we used primary breast tumor data [2,11,12] that is publicly available from the Stanford Microarray Database and the Gene Expression Omnibus. DOX-treated patients for which p53 status had been determined by sequence analysis [2] were included in our analysis (102 tumor samples, including 8 normal-like breast samples, one unclassified tumor, and 37 before and after pairs, representing 69 patients in total). All tumor subtypes described in Sorlie *et al*. [2] [classified using intrinsic analysis [12]] were included, except true normal breast and normal-like breast tumor samples. This sample set also included tumors collected before and after treatment with doxorubicin. The gene expression patterns of the p53 mutant samples (n = 43) were compared to those of the p53 wildtype samples (n = 52).

### Identification of p53 functional status in independent test data sets

A final 52 gene list was derived by identifying those genes that were differentially expressed in response to p53 loss in both the *in vitro *and *in vivo *data sets. These genes were matched to publicly available array data [13,14], using unique Unigene identifiers. Of the 52 genes, 48 and 50 were present on the Chang *et al*. data set and Miller *et al*. data sets, respectively. Microarray platform/source systematic biases between the training and the test sets were corrected using Distance Weighted Discrimination (DWD) [15]. To classify tumors in the independent test sets (Chang *et al*. or Miller *et al*.) as p53-functional or not, two centroids were created using the Sorlie *et al*. training set. The centroids were based on average gene expression in tumors in Figure 5A (mutant enriched) vs. that of tumors in Figure 5B (wildtype enriched). Each Chang *et al*. or Miller *et al*. tumor was classified according to the nearest centroid as determined by Spearman correlation.

### Other statistical analyses

Survival analyses were conducted using Sorlie *et al*. tumor data (excluding duplicate samples from the same person, resulting in a total of 66 patients representing 31 disease-specific and 26 overall survival events for survival analyses), Change *et al*. tumor data [337 patients: 295 patients from [13] and 42 tumors published in an earlier paper [16] from the same group, representing 126 disease specific and 79 overall survival events] and Miller *et al*. [14] data (236 patients, 52 disease specific events). For analyses of the Miller *et al*. dataset, patients that had survived at least ten years were censored to be consistent with previous analyses [14]. Kaplan Meier analyses were conducted using WinStat for Microsoft Excel.

Because the large data set of Chang *et al*. also included data on other prognostic variables, Cox proportional hazards modeling was conducted (SAS version 9.1). The reduced model that included ER status (positive vs. negative), tumor size (≤ 2 cm vs. > 2 cm), lymph node status (indicator coding with three categories: 0, 1–3, > 3 positive nodes or metastatic), age (in decades), grade (indicator coding with three categories: 1, 2, 3), and treatment (yes if treatment with chemo and/or hormonal therapy, no if no adjuvant therapy) was compared to a full model that also included a binary variable indicating p53 classification (based on gene-expression).

To determine if p53 status differed according to tumor subtype, a Fisher-Freeman-Halton (FFH) exact test was conducted using SAS version 9.1 (Cary, NC). Analyses of sequence-based mutation characteristics (e.g. missense/in-frame vs. nonsense and frameshift, missense DNA binding vs. non-DNA binding) in association with gene expression classification were also conducted using FFH exact tests.

## Results

### Gene expression and phenotypic analysis of cell lines expressing p53 RNAi

**Figure 1:**
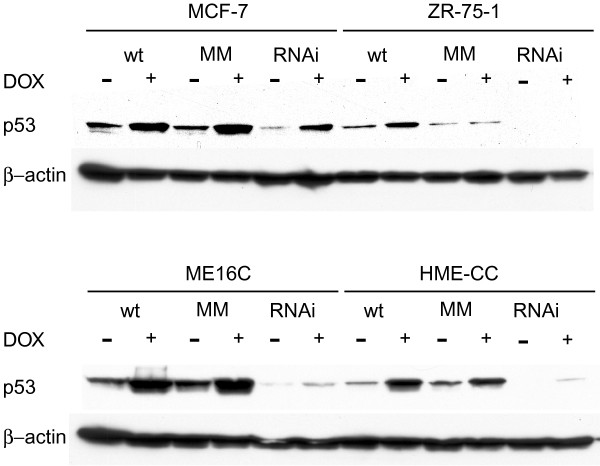
**p53 protein expression is knocked down by RNAi expression**. The expression of p53 was examined by Western blot analysis of extracts from MCF-7, ZR-75-1, HME-CC, and ME16C cell line parents and the same cell lines stably transduced with p53-targeted RNAi vector or p53 mismatch (MM) RNAi vector. Treatment with doxorubicin (24 h, 1μM) induced p53 expression in all cell lines and transductants, but induced levels were markedly lower in the p53-RNAi cells.

To study the effects of p53 loss *in vitro*, an RNAi construct specific for p53 [6] was stably expressed in MCF-7, ZR-75-1, ME16C and HME-CC cells. All four cell lines had wildtype p53 sequence and expressed functional p53 (showed p53 induction in response to treatment with DOX, Figure 1) prior to transduction with the p53-RNAi retroviral construct. Expression of p53-RNAi substantially knocked down p53 protein levels in both treated and untreated cells (Figure 1).

**Figure 2:**
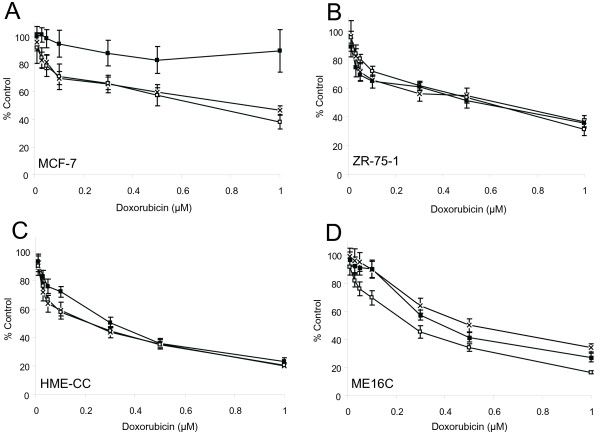
**Chemosensitivity is altered in the MCF-7 cell line following transduction with p53-RNAi**. ME16C, HME-CC, and ZR-75-1 cell lines had similar chemosensitivity curves for wildtype (x), p53-targeted RNAi expressing cells (solid square) and p53 mismatch RNAi expressing cells (open square). Only the p53-RNAi MCF-7 cells showed significant chemoresistance.

**Figure 3:**
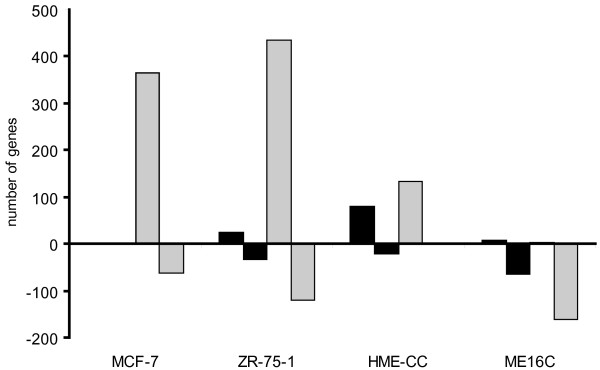
**Number of genes with significantly different expression following p53 loss (by RNAi) in sham-treated (black) or doxorubicin-treated (gray) cells**. Sham-treated cells represented feeding controls, treated with fresh media and harvested at the same time points as the doxorubicin treated cells. Positive y-axis indicates number of genes up-regulated and negative y-axis indicates number of genes down-regulated by p53 knockdown. Luminal cell lines had the largest transcriptional response to p53 loss following induction, while HMEC cell lines had a stronger signature for p53 loss in the uninduced state.

The phenotypic effects of p53 knock-down varied by cell line (Figure 2). MCF-7 cells became more resistant to DOX, while ZR-75-1, ME16C and HME-CC cells displayed no change in DOX sensitivity. Consistent with the different responses in the DOX sensitivity assay, gene expression signatures significantly associated with p53 loss (in 2-class SAM analyses) were different for each cell line and cell type (gene lists are given in Additional File 1). As shown in Figure 3, MCF-7 and ZR-75-1 cells showed a stronger p53-dependent signature following treatment with DOX. The immortalized HMECs, conversely, showed stronger p53-dependent signatures in the absence of DOX (i.e. parents vs. RNAi, both untreated). Analysis of SAM-derived gene lists using gene ontology software (EASE) showed enrichment for categories of genes with known relevance to p53 function. For example, among the DOX-treated samples (DOX-treated parent vs. DOX-treated RNAi-expressing), three cell lines (HME-CC, MCF-7 and ZR-75-1) increased genes involved in mitosis after transduction with p53-RNAi. ME16C did not induce categories of mitosis genes, but did suppress negative regulators of cell proliferation. Significant down-regulation of apoptotic genes was only seen in ZR-75-1 cells.

The p53-response observed among DOX-treated cell lines differed from the p53-response in sham-treated cell lines. For example, the luminal-like cell lines (MCF-7 and ZR-75-1) that had the largest transcriptional response to DOX, showed a modest response to p53 loss in sham-treated samples (sham-treated parent versus sham-treated RNAi-expressing). Sham-treated MCF-7 cells showed no significant changes and ZR-75-1 cells showed few changes in response to p53 loss. EASE analysis of the ZR-75-1 changes did not identify categories with clear relevance to p53 signaling. Only one down-regulated gene ontology category (extracellular region) was identified. Induced gene categories were transition metal homeostasis genes and genes with unknown roles in biological processes. However, among the basal-like cell line models, ME16C significantly down-regulated anti-apoptosis genes and HME-CC significantly up-regulated mitosis/proliferation genes. The strong mitotic signature of sham-treated HME-CC cells showed overlap with the strong mitotic signature observed in DOX-treated HME-CCs. Thus, p53 loss had different effects across cell type and cell line.

Common patterns of expression shared by most of the four lines were identified using a 2-class SAM (DOX- and sham-treated combined from all parental lines vs. all p53-RNAi expressing lines) to analyze all four cell lines simultaneously. In addition to identifying a common response, this analysis had a larger sample size and thus, had better power to detect a broader range of p53-regulated genes. There were 696 genes which responded significantly to p53 loss in the cell lines (Additional File 2). Included in this list were many known direct p53 targets including MDM2, p21 (Cip1), GADD45A, and ribonucleotide reductase M2. All of these genes had lower expression in p53-RNAi lines, consistent with expectation. In total, 357 of the 696 significantly altered genes had lower expression in p53-RNAi lines; EASE analysis indicated that apoptosis genes, cell death genes, and regulators of programmed cell death were significantly over-represented. Conversely, there were 339 genes (of 696 significantly altered genes) that were more highly expressed in RNAi lines, including genes involved in mitosis, cell cycle control, and regulation of DNA repair.

### Gene expression signatures of primary tumors with wild-type and mutant p53

**Figure 4:**
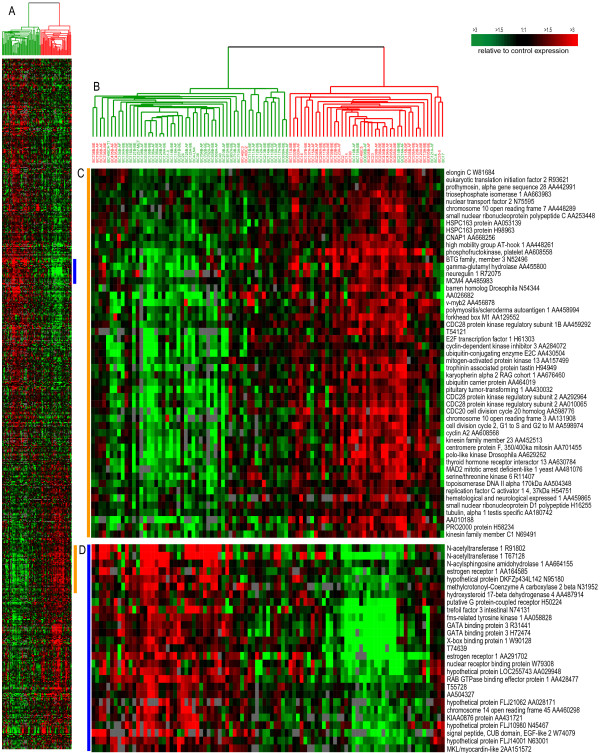
**Gene expression pattern of tumor samples for 747 genes correlated with p53 status in tumors**. The fold change relative to the median expression value across all tumors is shown. Colored bars in A illustrate the location of clusters C and D. The dendrogram in B shows that experiments were divided into two primary branches, one enriched for mutant (red) and one enriched for wildtype (green) tumors. Tumor sample names are red for mutants and green for wildtypes based on sequence analysis. Clusters enriched for proliferation and cell cycle genes (C) and for luminal/estrogen responsive tumor markers (D) are shown.

Gene expression data for primary breast tumors with known p53 mutation status is publicly available [2,12]. Using this data, we found that the expression of 747 genes was significantly correlated with p53 status (Figure 4A). The hierarchical cluster of these genes across the primary tumors contained two branches (Figure 4B), one enriched for wild-type tumors (left branch, 45 of 53 wildtype samples) and one enriched for mutant tumors (right branch, 34 of 42 mutant samples). A proliferation cluster/signature was differentially expressed across the two branches of the dendrogram (Figure 4C). This cluster had higher expression in p53 mutants, and included the cell cycle associated genes cyclin A2, CDC28 subunit 1B, CDC2, cyclin-dependent kinase inhibitor 3, polo-like kinase, and topoisomerase IIA. EASE analysis confirmed that genes involved in mitosis and cell cycle progression were significantly over-represented in the set of genes that had higher expression in p53 mutant tumors.

A cluster (Figure 4D) enriched for genes associated with the luminal/ER+ tumor subtypes (N-acetyltransferase 1, estrogen receptor 1, putative G-protein-coupled receptor, trefoil factor 3, GATA binding protein 3, and X-box binding protein 1) was also present in this gene set [2,11,12]. This cluster was more highly expressed in wildtype tumors, likely due to a larger representation of luminal tumors in this branch. In fact, when the intrinsic subtype of each of the patients in Figure 4 was determined by clustering all 95 tumor samples using the intrinsic list of Sorlie *et al*. [12], a statistically significant association between p53 status and tumor subtype was observed (p = 0.002), with 31% of luminal tumors and 80% of basal-like tumors having mutant p53. Because the frequencies of p53 status varied significantly by subtype, the list of p53-associated genes defined by SAM includes genes that were associated with subtype. Some of these genes may have no causal association with p53 defects, and thus, refinement of this list using our *in vitro *data was performed.

### Combined *in vitro *and *in vivo *analysis to identify p53-regulated genes

The *in vitro *experiments that we conducted contained isogenic pairs of cell lines that were representative of both luminal and basal-like tumors. The *in vivo *experiments represented tumors derived from 69 different individuals, also representing both luminal and basal-like tumors. By comparing the p53-associated gene lists from the tumors to the cell lines, we refined our gene list and obtained a list of genes that were common to both data sets, representing a stereotypic p53 signature that held across diverse genetic backgrounds. There were 52 genes that were identified in common between the *in vivo *(747 genes) and *in vitro *(696 genes) lists. This 52-gene list retained GATA binding protein 3 and many of the proliferation cluster genes in Figure 4C (ATPase Family AAA domain containing 2, gamma-glutamyl hydrolase, MYBL2, CDC28 subunit 1B, CDC2, cyclin A1). However, this list excluded ER and many of the luminal tumor-associated genes shown in Figure 4D. This list still contains a few p53-regulated genes that are also ER associated (such as GATA3), however their presence on this list cannot be viewed as an artifact of their association with ER status.

**Figure 5:**
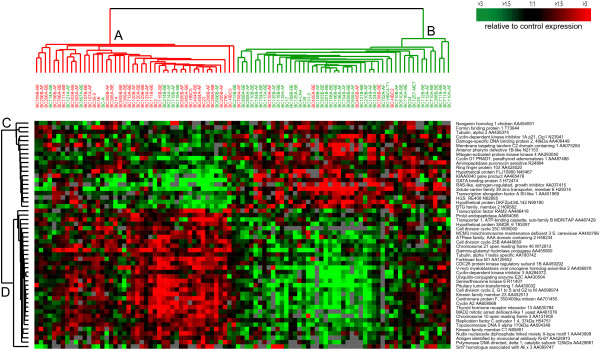
**Cluster analysis of the tumor samples using 52 genes correlated with p53 status in tumors and cell lines**. The fold change relative to the median expression value across all tumors is shown. The red dendrogram branch (A) is enriched for p53 mutant tumors (sample names labeled red) while the green dendrogram branch (B) is enriched for p53 wild-type tumors (sample names labeled green). The dendrogram showing the gene clusters is shown in C and D.

Patterns of expression for these 52 genes are shown across the primary tumor data in Figure 5. Again, two dendrogram branches were evident: one enriched for p53 mutants (Figure 5A) and the other enriched for p53-wildtypes (Figure 5B). Figure 5 also shows two main clusters of genes, one of which (Figure 5C) was enriched for genes that are known to be p53-regulated including p21 (Cip1), BTG2, and damage-specific DNA binding protein 2. EASE analysis confirmed that this cluster, which had lower expression in mutant tumors, contained DNA damage response genes and negative regulators of cell proliferation. The second gene cluster (Figure 5D) was more highly expressed in mutant tumors, and EASE analysis confirmed that this cluster of genes was enriched for mitosis and proliferation genes.

### Survival analyses using the 52-gene p53 signature

**Figure 6:**
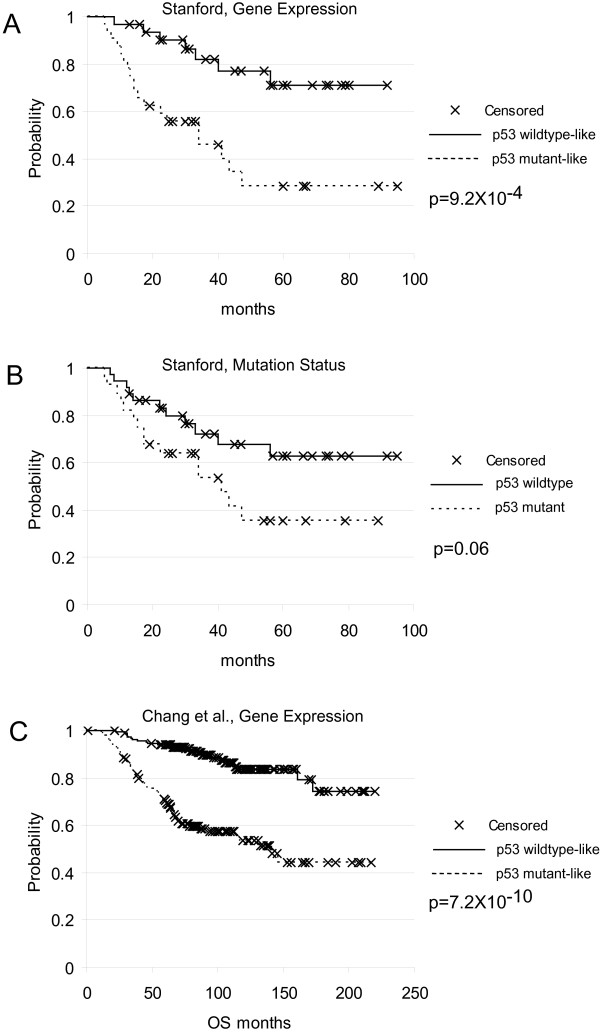
**Kaplan-Meier survival curves for patients with p53 wildtype (solid line) or mutant (dashed line) tumors using gene expression-based p53 functional category or mutation status**. Overall survival analysis comparing the two gene-expression based dendrogram groups (tumors in Figure 5A vs. tumors in Figure 5B) yielded a highly significant difference in survival, which compares favorably with mutation status. Both analyses used 66 tumor samples and included 26 events. Survival analysis on a separate data set (Chang et al., 2005) including 337 tumors and 79 events (C) also yielded highly significant differences based on gene expression classification.

Kaplan-Meier survival analysis yielded highly significant survival differences between groups from Figure 5A (mutant-like) and 5B (wildtype-like) using the Sorlie *et al*. data. As shown in Figure 6, the 52-gene expression signature (p = 0.001) significantly predicted overall survival (OS), while true mutation status on this set of samples was not significant (p = 0.06). The expression signature (p = 2.2 × 10^-5^) and true mutation status (p = 0.001) also significantly predicted relapse-free survival (RFS). To further evaluate the prognostic value of this 52-gene signature, we performed survival analyses using two independent breast tumor data sets [published by Chang *et al*. (2005) and Miller *et al*. (2005)]. Kaplan-Meier analysis showed that this signature significantly predicted OS (Figure 5C, p = 7.2 × 10^-10^) and RFS (p = 2.6 × 10^-7^) for the Chang *et al*. dataset, and disease-specific survival (p = 0.007, RFS and OS data unavailable) for the Miller *et al*. dataset. We also performed multivariate analysis, using the Chang *et al*. dataset. Controlling for standard clinical predictors (ER, grade, node status, size, age, and treatment), Cox proportional hazards ratios were estimated for both OS (RH, 95% CI: 2.4, 1.3 – 4.4) and RFS (RH, 95% CI: 1.9, 1.2 – 3.0). Thus, independent of standard clinical predictors the p53 expression classifier significantly predicted both OS (p = 0.006) and RFS (p = 0.006).

In our training data set (Sorlie *et al*.), the gene expression classifier had 82% agreement with sequence-based mutation status. True mutation status data was not available for the Chang et al. data set, but our classifier had 82% agreement with sequence-based mutation status in the Miller *et al*. data set. We were able to examine the location and type of mutations and compare them to classifier results using the Miller et al. data. Of the 29 mutants incorrectly classified as wildtype, 25 (86%) were either missense mutations or in-frame insertions/deletions. This differs significantly (p = 0.02) from the percentage of mutations that were missense or in-frame among correctly classified mutants (58%). Among the missense tumors, mutations in DNA binding domains of the p53 protein were also significantly more frequent (p = 0.01) in tumors classified as mutant (87%) than wildtype (45%).

## Discussion

Identification of a p53-responsive signature in breast cancer is confounded by associations with important tumor characteristics like ER status. The common p53 expression signature shared by cell lines and tumors in this study addressed this confounding by conducting cell lines experiments with ER positive and ER negative cell lines, and using experimental data to refine the gene lists derived from observational studies in patients. The resulting 52 gene, p53-associated list contained two biologically relevant gene clusters corresponding to downregulated and upregulated genes. This finding is consistent with the previous literature showing that p53 transactivates genes such as p21 and GADD45 and transrepresses genes such as topoisomerase IIA and CDC2. Inactivation of p53 affects both transactivation and transrepression to alter cell growth. Inactivation of p53 is also likely to cause downstream, indirect effects. As more research is conducted to identify pathway signatures [17,18], evidence is growing that most, if not all, pathway signatures include both direct and indirect targets. However, these signatures still appear to show pathway-specific activity and represent valuable assays for pathway activity [19]. So, while we cannot conclude that these are exclusively direct targets of p53, genes in our signature do represent a common response to p53 loss in the breast.

This common p53-response list is biologically relevant, as shown by its ability to predict survival in patients across multiple true test data sets. Some of the genes in the common expression profile have been previously identified in other signatures of prognostic relevance (e.g. proliferation-associated genes) and are likely to be regulated by multiple oncogenic pathways. Our aim was not to identify a new prognostic signature that improves on previously published signatures. Rather, we aimed to demonstrate that events that are downstream of functional p53 loss are clearly associated with prognostic outcome, and are therefore biologically relevant. The predictive accuracy of p53-dependent gene expression profiles [14] supports a role for p53 in breast cancer prognosis. Previous estimates of the relative hazard (RH) associated with p53 loss range from 1 (no effect) to 23 [20]. Our data suggests that this variability may relate to limitations of the methods for characterizing p53 status. p53 mutation status is most commonly characterized by direct DNA sequencing or by immunohistochemistry (IHC). Sequencing analysis cannot distinguish sequence variants with and without functional consequences. A meta-analysis of p53 mutation databases has demonstrated methodological biases associated with sequence-based mutation status [21]. IHC analysis treats accumulation of p53 protein as indicative of mutation; thus, IHC is biased toward identification of missense mutants and completely misses mutations that cause loss of p53 protein. With either IHC or sequence analysis, a narrow emphasis on p53 mutations can miss functional impairments in the p53 pathway (e.g. MDM2 amplification). These challenges could account for widely divergent estimates of p53's role in prognosis.

Our data analysis also showed that there was good agreement between mutation status and expression profiles. Using our 52 gene list, there was >80% agreement between p53 mutation status and p53 expression class in both the Sorlie *et al*. and Miller *et al*. datasets. This high level of agreement across data sets attests to the fact that the signature is indicative of p53 across a wide range of cell backgrounds. If the signature were merely correlated with proliferation, ER status, or another tumor characteristic, then poor concordance with p53 mutational status would be expected in cross validation. The samples where gene expression and mutation status disagree may represent true differences in the functional p53 pathway. For example, the tumor BC606 was p53 wildtype by sequence but clustered with p53 mutants using the 52 gene classifier. This tumor overexpressed MDM2 mRNA (data not shown), a key negative regulator of p53. Among the false negatives (sequence mutant but wildtype expression signature), our analysis of the Miller et al. data showed that misclassified mutants had higher proportions of mutation types that are likely to be less deleterious (missense mutations and in-frame insertion/deletions).

In addition to identifying a stereotyped signature associated with p53 loss, these results demonstrate that the relative importance of p53-regulated functions such as cell cycle control, DNA repair, and apoptosis are subject to significant inter-individual variation. Each cell line displayed a unique p53 response signature. However, similarities according to cell type were also evident. Both of the HMEC-derived cell lines showed a greater response to p53 loss in the untreated state, while the MCF-7 and ZR-75-1 lines showed a stronger p53-regulated signature following DOX treatment. These results extend previous observations [5] suggesting a difference in p53 signaling pathways between luminal and basal-like breast cancers. These inherent differences in p53 signaling could lead to different selection pressure for p53 loss in each cell type. Such differences could also explain the divergent rates of p53 mutation by subtype that have been reported here and in a population-based study [3].

Our data reconfirmed the complex relation between chemosensitivity and p53 status [22]. Previous reports have demonstrated either heightened chemosensitivity of p53 mutants [23,24] or heightened chemoresistance [25]. This paradox is reflected in our study where the four cell lines we studied varied widely in their DOX sensitivity following p53-knockdown. Because p53 regulates many different pathways, including DNA repair, apoptosis, and cell proliferation, and the balance of these various pathways determines chemosensitivity, it is not surprising to find that both individuals and cell lines have responses to chemotherapy that are difficult to predict. DOX also has many p53-independent toxicity mechanisms, so a divergence in sensitivity across lines may also reflect differences in how DOX toxicity is manifest across lines. These analyses have demonstrated that breast cell lines have individual, distinct responses to p53 loss. The genetic background of a given cell line, including cell type of origin, plays a prominent role in mediating p53 signaling.

A strength of our study was the use of cell line experiments to control a range of variables that influence p53-response. The *in vitro *setting allowed for control of expression of p53 protein, breast cancer subtype, and p53-inducing events [26-29]. However, the *in vitro *approach is limited in that a small number of cell lines can be reasonably examined, representing only a handful of tumors. By combining the *in vitro *expression data with data from human tumors assayed before and after DOX treatment, we examined a much wider range of individual responses to p53 loss than cell line experiments could reasonably examine, and performed a controlled experiment that cannot be accomplished in humans. Previous studies have characterized p53-responses in breast cancer using gene expression data from tumors and statistical models to try to control the effects of breast cancer heterogeneity. For example, in Miller et al. [14], proliferation and ER status were treated as statistical confounders of the p53-gene expression relation (based on p53 status and outcome both having crude associations with grade and ER status). Thus, the final p53-mutant like gene expression profile presented by Miller et al. [14] was derived using a statistical model that adjusted for these variables. Such adjustment assumes that grade and ER status are causally upstream of p53 status. If grade and ER status are downstream of p53 status, this approach will introduce a bias toward exclusion of grade and ER-associated genes, even though those genes are influenced by p53 loss. In short, the validity of statistical adjustment depends upon having the correct model for the relation between breast cancer subtype, ER status, proliferation and p53 biology. In the presence of heterogeneity, experimental and biologically-based methods for assessing gene expression in relation to p53 status are preferable to statistical methods.

Many of the genes associated with p53 loss in this analysis were of prior interest in breast cancer. For example, GATA3 is involved in growth control and maintenance of the differentiated state in breast epithelial cells and has been hypothesized to play a role in tumorigenesis of ER-positive breast tumors [30]. p21 (Cip1), CDC2, and CDC25C are genes involved in p53-mediated regulation of cell cycle arrest [31]. Pituitary tumor-transforming 1 is a recently identified oncogene with p53-dependent and p53-independent functions [32]. Thus, as might be expected, many of the direct and indirect targets of p53 identified here are known p53- and cancer-associated genes. Further investigation of the specific p53 targets that are regulated in common across breast cancers and investigation of those that are differentially regulated across breast cancer subtypes will add to our understanding of the biology of breast cancer and breast cancer subtypes.

## Conclusion

In the presence of breast cancer heterogeneity, controlled experiments *in vitro *combined with *in vivo *analyses, allowed for refinement of a p53-associated gene set. The refined 52-gene list excluded genes that were associated with breast cancer subtype and not downstream of p53. This work identified a signature for p53 loss that is shared across breast cancer subtypes and that provided prognostic information and a biologically-relevant gene set.

## Competing interests

The author(s) declare that they have no competing interests.

## Authors' contributions

MAT participated in study design, created the isogenic cell line pairs, performed toxicity assays, western blots, and microarrays, performed analysis and interpretation, drafted and revised the manuscript. JIH participated in study design, creation of cell line pairs, interpretation of results, and manuscript revisions. DSO performed survival and gene ontology analyses. XH performed microarrays. KAH participated in toxicity assays and manuscript revisions. CSB participated in interpretation and critical review of the manuscript. CP participated in study design and coordination, supervision of experimental conduct and analysis, interpretation of results, drafting and revision of the manuscript, and approved the final version. All authors have read and approved the final manuscript.

## Pre-publication history

The pre-publication history for this paper can be accessed here:



## Supplementary Material

Additional file 1 Microsoft Excel spreadsheet (196 kB) containing lists of genes for which expression levels were significantly associated with p53 loss (in 2-class SAM analyses). Click here for file

Additional file 2 Cluster figure showing parents and RNAi-transductants for all four parent cell lines (MCF-7, ZR-75-1, HME-CC, and ME16C). Genes included are the 696 genes identified as significant by SAM analyses comparing parents to RNAi-transductants. Click here for file
