# Supplementary material for: Gene expression patterns associated with p53 status in breast cancer
Source: BMC Cancer. 2006 Dec 6;6:276. doi: 10.1186/1471-2407-6-276 (PMC1764759; doi:10.1186/1471-2407-6-276)

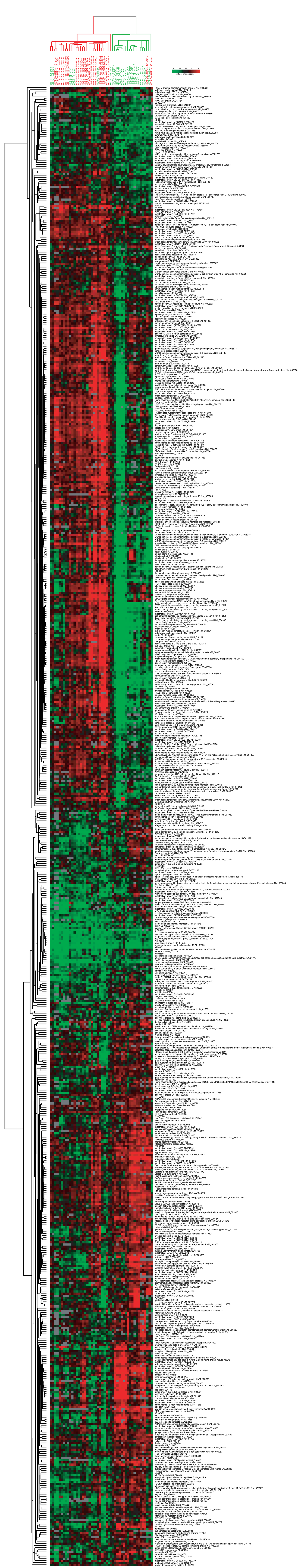

Supplement: Additional file 2 — Cluster figure showing parents and RNAi-transductants for all four parent cell lines (MCF-7, ZR-75-1, HME-CC, and ME16C). Genes included are the 696 genes identified as significant by SAM analyses comparing parents to RNAi-transductants. [file 1471-2407-6-276-S2.pdf]
